# Psychiatric disorders in incident patients with juvenile idiopathic arthritis - a case-control cohort study

**DOI:** 10.1186/s12969-021-00599-x

**Published:** 2021-07-02

**Authors:** Minna S. Kyllönen, Hanna Ebeling, Hannu Kautiainen, Kari Puolakka, Paula Vähäsalo

**Affiliations:** 1 PEDEGO Research Unit, University of Oulu, Oulu, Finland; 2 Department of Internal Medicine, Oulu University Hospital, Oulu, Finland; 3 Medical Research Center Oulu, Oulu University Hospital and University of Oulu, Kiviharjuntie 9, 90220 Oulu, Finland; 4 Department of Child Psychiatry, Oulu University Hospital, Oulu, Finland; 5 Department of General Practice and Primary Health Care, University of Helsinki, Helsinki, Finland; 6 Terveystalo Healthcare, Lappeenranta, Finland; 7 Department of Pediatrics, Oulu University Hospital, Oulu, Finland

**Keywords:** Juvenile idiopathic arthritis, Psychiatric disorders, Chronic illness, Mental health

## Abstract

**Background:**

Chronic illness, such as juvenile idiopathic arthritis (JIA), appears to have an impact on the mental health of children and adolescents. The aim of this study was to explore the incidence of mental and behavioural disorders according to age at JIA onset and gender in JIA patients compared to a control population.

**Methods:**

Information on all incident patients with JIA in 2000–2014 was collected from the nationwide register, maintained by the Social Insurance Institution of Finland. The National Population Registry identified three controls (similar regarding age, sex and residence) for each case. They were followed up together until 31st Dec. 2016. ICD-10 codes of their psychiatric diagnoses (F10-F98) were obtained from the Care Register of the National Institute for Health and Welfare. The data were analysed using generalized linear models.

**Results:**

The cumulative incidence of psychiatric morbidity was higher among the JIA patients than the controls, hazard ratio 1.70 (95% Cl 1.57 to 1.74), *p* < 0.001. Phobic, anxiety, obsessive-compulsive, stress-related and somatoform disorders (F40–48) and mood (affective) disorders (F30–39) were the most common psychiatric diagnoses in both the JIA patients (10.4 and 8.2%) and the control group (5.4 and 5.1%), respectively. Female patients were more prone to mental and behavioural disorders than males were, and the risk seemed to be higher in patients who developed JIA in early childhood or adolescence.

**Conclusion:**

Patients with JIA are diagnosed with mental and behavioural disorders more often than controls, and the age at onset of JIA could have implications for future mental health.

## Background

Juvenile idiopathic arthritis (JIA) is a heterogeneous inflammatory rheumatic disease with onset before the age of 16 years. It is classified into seven categories according to the ILAR (International League of Associations for Rheumatology) criteria [1] .

Children and adolescents with JIA are more likely to have psychiatric problems than their healthy counterparts are [2–7]. Varying results have been reported, mostly due to differences in study populations (age, disease state/disability, disease duration, medication) and differences in study methods such as self-questionnaires [4–15], psychiatric interviews [2, 3] or quality of life studies [4, 5, 16–21]. Using questionnaires, some studies have found no differences in mental health or psychiatric disorders between patients with JIA and controls [14–20, 22, 23], whereas other studies have reported significant differences [4–7].

The most common psychiatric disorders in both JIA patients and the general population are depression and anxiety [2, 3, 24, 25], and most questionnaire-based studies have focused on these disorders [4–15]. Only a few have reported increased numbers of behavioural problems in JIA patients [7, 11, 15, 23].

To our knowledge, there are no studies exploring the whole spectrum of clinical mental and behavioural disorders among patients with JIA. Most of previous studies have focused on depression, anxiety and behaviour problems through self-questionnaires and on mental health from a quality of life perspective. In this study, we explored all psychiatric diagnoses in JIA patients diagnosed between 2000 and 2014 and compared their psychiatric morbidity with that of the control population. In our extensive data we also studied the influence of gender and the child’s age at JIA onset on psychiatric morbidity.

## Patients and methods

In Finland, patients with certain chronic and severe diseases, such as JIA, are entitled to a special (higher) reimbursement of medication costs from the Social Insurance Institution (SII). From this national reimbursement register, information on all patients with the ICD-10 code of M08.0-M08.9 or M09.0*L40.5 and with the date of first reimbursement decision from 1st Jan. 2000 to 31st Dec. 2014 (index date) were collected. Mild cases of JIA with no need for disease-modifying antirheumatic drug were not included in the SII register. In this study, the index day serves as a surrogate of the day of diagnosis. For each case, the National Population Registry identified three controls individually matched for age, sex and residence at the index date. The individuals were followed up until 31st Dec. 2016. The data were de-identified.

In Finland, municipalities are responsible for organizing health services in their area, including primary health care and hospitals for special outpatient and inpatient care. The Finnish law on the personal registers obligates the service providers to produce information to the Care Register of National Institute for Health and Welfare. The Care Register covers all hospital care in Finland since 1969, and since 1998, secondary care outpatient visits have been included. Information includes personal identification code and diagnoses of the patient’s medical problems as codes of the International Classification of Diseases 10th Edition (ICD-10). Psychiatric diagnoses (ICD-10 codes of F10–98) since the index day were obtained from the Care Register.

Psychiatric diagnoses were grouped into eight main categories (Table 1). Mental retardation (F70–79) was not included in the analysis.

**Table 1:** Psychiatric diagnoses and International Classification of Diseases codes (ICD-10)

Psychiatric Diagnoses	ICD-10
**Mental and behavioural disorders due to psychoactive substance use**	**F10–19**
**Psychotic disorders**	**F20–29**
**Mood (affective) disorders**	**F30–39**
**Mania and bipolar disorders**	
**Depression and other mood disorders**	
**Neurotic, stress-related and somatoform disorders**	**F40–48**
**Phobic and anxiety disorders**	
**Obsessive-compulsive disorder**	
**Reaction to stress and adjustment disorders**	
**Dissociative disorder**	
**Somatoform disorder**	
**Others**	
**Behavioural syndromes associated with physiological disturbances and physical factors**	**F50–59**
**Eating and sleeping disorders**	
**Psychological and behavioural factors associated with disorders or disease**	
**Others** ^**a**^	
**Disorders of adult personality and behaviour**	**F60–69**
**Disorders of psychological development**	**F80–89**
**Childhood behavioural and emotional disorders**	**F90–98**
**Hyperkinetic and conduct disorders**	
**Emotional disorders specific to childhood onset**	
**Disorders of social functioning, tic disorders and others**	

^a^ Sexual dysfunction, behavioural disorders associated with puerperium, abuse of non-dependence-producing substances

### Statistical methods

Cumulative incidence of the first psychiatric diagnoses was based on the product limit estimate (Kaplan-Meier) of cumulative function. The Cox proportional hazard model was used to estimate the psychiatric morbidity risk of the JIA patients and the control population. The results are presented as hazard ratios (HR) with 95% confidence intervals (CIs). The number and incidence were calculated assuming a Poisson distribution. Incidence rate and incidence rate ratios (IRR) were calculated using a Poisson regression model. The Poisson regression was tested using the goodness of fit of the model, and the assumption of overdispersion in the Poisson model was tested using the Lagrange multiplier test. A possible nonlinear relationship between incidences of psychiatric diagnoses and the age at diagnosis were assessed using a 4-knot-restricted cubic spline Poisson regression model. Stata 16.0 (Stata Corp LP, College Station, TX, USA) was used for the analyses.

Permission to use the databases was obtained from the SII and the National Institute for Health and Welfare. According to Finnish legislation, no ethical committee approval or patients’ informed consent is required for register-based studies done without contacting study subjects.

## Results

### Patient characteristics

A total of 4180 JIA patient (2603 females, 1577 males) with the index day during 2000–2014 were identified. The mean age (SD) at the index date was 8.3 (4.8) years. The median follow-up time was 6.6 years (IQR 3.1–10.5). The mean age at the end of follow-up was 14.8 (6.4) years. The patients were compared with 12,512 population controls, matched for age, sex and residence at the index date (7793 females, 4719 males). The total follow-up time was 37,239 person years in JIA patients and 111,684 person years in controls.

### Incidence of mental and behavioural disorders

**Fig. 1:**
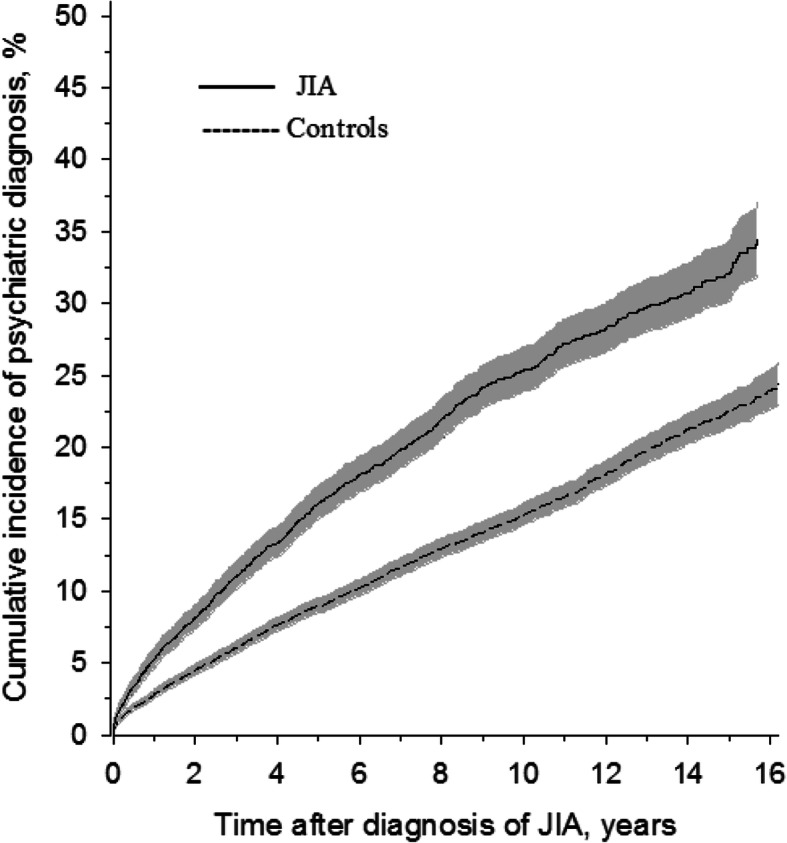
Kaplan-Meier-estimated psychiatric morbidity of the patients with incident juvenile idiopathic arthritis (JIA) and controls. Grey area represents 95% confidence intervals

During the follow-up time, 959 (22.9%) JIA patients and 1787 (14.3%) comparators were diagnosed with some mental or behavioural disorder. The risk of developing psychiatric morbidity was higher among the JIA patients, HR 1.70 (95% Cl 1.57 to 1.74), females’ HR 1.83 (1.66 to 2.01), males’ HR 1.49 (1.30 to 1.71). The 10-year cumulative incidence was 25.3% (95% Cl 23.8 to 26.9) in JIA patients and 15.3% (95% Cl 14.6 to 16.0) in controls (Fig. 1).

**Fig. 2:**
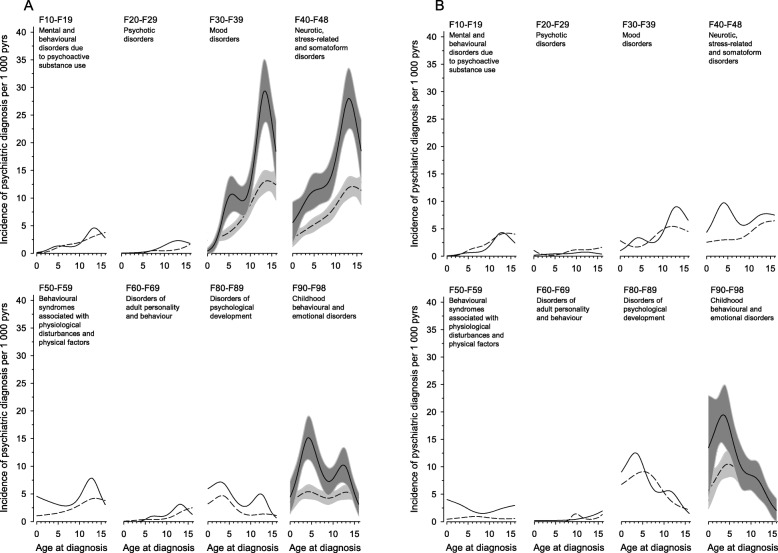
Incidence rate of psychiatric diagnoses in juvenile idiopathic arthritis patients and controls, separated by gender. Incidence rate of psychiatric diagnoses in juvenile idiopathic arthritis (JIA) patients and controls according to age in years at JIA onset in eight main psychiatric diagnostic categories (F10-F98), females (A) and males (B). Incidence rate of JIA is indicated with a solid line and incidence rate of controls with a dashed line. Grey area represents 95% confidence intervals

We analysed psychiatric disorder incidence rates in diagnosis groups by gender and age at JIA diagnosis comparing patients with controls. The two most common diagnosis groups were neurotic, stress-related and somatoform disorders (F40–48) and mood disorders (F30–39) both in JIA patients and in controls, and the incidence rate was higher among females (Table 2 and Fig. 2). Childhood behavioural and emotional disorders (F90–98) was the third in frequency. Here the gender difference was minor.

**Table 2:** Incidence of psychiatric diagnoses per 1000 person years (pyrs) and incidence rate ratios (IRRs) in juvenile idiopathic arthritis (JIA) patients compared to controls

Psychiatric Diagnoses (ICD-10)	JIA (*n* = 4180) n (%)	JIA Incidence/1000 pyrs (95% Cl)	Controls (*n* = 12,512) n (%)	Controls Incidence/1000 pyrs (95% Cl)	IRR (95% Cl)
Mental and behavioural disorders due to psychoactive substance use (F10–19)					
Females	46 (1.10)	2.00 (1.47, 2.67)	123 (0.98)	1.78 (1.48, 2.13)	1.12 (0.80, 1.57)
Males	24 (0.57)	1.72 (1.10, 2.56)	89 (0.71)	2.13 (1.71, 2.62)	0.81 (0.52, 1.26)
Psychotic disorders (F20–29)					
Females	24 (0.57)	1.04 (0.67, 1.55)	33 (0.26)	0.48 (0.33, 0.67)	2.19 (1.29, 3.70)
Males	7 (0.17)	0.50 (0.20, 1.03)	35 (0.28)	0.83 (0.58, 1.16)	0.60 (0.27, 1.35)
Mood (affective) disorders (F30–39)					
Females	279 (6.67)	12.86 (11.39, 14.46)	493 (3.94)	7.33 (6.70, 8.00)	1.75 (1.52, 2.03)
Males	63 (1.51)	4.59 (3.52, 5.87)	149 (1.19)	3.59 (3.04, 4.22)	1.28 (0.85, 1.71)
Neurotic^a^, stress-related and somatoform disorders (F40–48)					
Females	335 (8.01)	15.50 (13.89, 17.25)	510 (4.07)	7.57 (6.93, 8.25)	2.05 (1.79, 3.45)
Males	100 (2.39)	7.33 (5.97, 8.92)	171 (1.37)	4.13 (3.54, 4.80)	1.77 (1.39, 2.27)
Behavioural syndromes associated with physiological disturbances and physical factors (F50–59)					
Females	100 (2.39)	4.42 (3.60, 5.38)	175 (1.39)	2.55 (2.19, 2.96)	1.73 (1.35, 2.22)
Males	34 (0.81)	2.44 (1.69, 3.41)	29 (0.24)	0.69 (0.46, 0.99)	3.55 (2.16, 5.83)
Disorders of adult personality and behaviour (F60–69)					
Females	26 (0.62)	1.13 (0.74, 1.65)	61 (0.49)	0.88 (0.67, 1.13)	1.28 (0.81, 2.02)
Males	8 (0.19)	0.57 (0.25, 1.12)	19 (0.15)	0.45 (0.27, 0.70)	1.26 (0.55, 2.88)
Disorders of psychological development (F80–89)					
Females	98 (2.34)	4.45 (3.62, 5.41)	169 (1.35)	2.47 (2.11, 2.87)	1.80 (1.40, 2.31)
Males	94 (2.25)	7.06 (5.71, 8.63)	245 (1.96)	6.04 (5.30, 6.84)	1.17 (0.92, 1.49)
Childhood behavioural and emotional disorders (F90–98)					
Females	202 (4.83)	9.16 (7.94, 10.51)	325 (2.61)	4.79 (4.28, 5.34)	1.91 (1.60, 2.28)
Males	145 (3.47)	10.88 (9.18, 12.81)	299 (2.39)	7.39 (6.58, 8.28)	1.47 (1.20, 1.80)

^a^ phobic, anxiety, obsessive-compulsive

When comparing patients with controls, females with JIA had higher incidence rate ratios than males with JIA, 1.71 (95% Cl 1.55 to 1.88) in females and 1.46 (95% Cl 1.28 to 1.66) in males. In females, IRRs were significantly elevated in all but two small diagnosis groups: mental and behavioural disorders due to psychoactive substance use (F10–19) and disorders of adult personality and behaviour (F60–69) (Table 2). Among males, IRRs were significantly increased in diagnosis groups of neurotic, stress-related and somatoform disorders (F40–48), behavioural syndromes associated with physiological disturbances and physical factors (F50–59) and childhood behavioural and emotional disorders (F90–98) (Table 2).

The age at which children were diagnosed with JIA was associated with incidence of mental and behavioural disorders. Incidence rate of behavioural disorders was highest among children who developed JIA in early childhood, whereas rates of neurotic, stress-related and somatoform disorders and mood disorders were highest among girls who developed JIA in adolescence (Fig. 2).

## Discussion

To our knowledge, this is the first large-scale longitudinal register-based study covering all mental and behavioural disorders in patients with JIA and the impact of the age at JIA onset on those disorders. We found that incident patients with JIA had a higher risk of mental and behavioural disorders than did the control population. The three most common disorders were neurotic, stress-related and somatoform disorders (F40–48); mood disorders (F30–39); and childhood behavioural and emotional disorders (F90–98). Some studies from recent years have reported results of same kind. Two cross-sectional questionnaire studies reported a higher incidence of behavioural problems, anxiety and depression in children and adolescent JIA patients compared to controls [6, 7]. More frequent psychiatric morbidity in patients with JIA was also observed in two small studies based on clinical assessment [2, 3]. However, some questionnaire studies did not show a difference in anxiety and depression between patients and controls [14–20, 22, 23].

We found that those who developed JIA in early childhood tended to have higher rates of behavioural disorders compared to controls than did those who were diagnosed with JIA in later years. In addition, anxiety and depression rates seemed to be higher in those patients with disease onset in adolescence than in those with onset in childhood (Fig. 2). According to psychological development theories, the onset of a chronic disease upsets the balance of mind and body [26], and the reaction to the traumatic experience depends on the age of the child [8, 26–28]. Children under the age of 7 years have limited ego functions, and they tend to react by expressing rage and aggression [11, 26], but by the primary school age, children have developed strategies to better handle emotional situations [26]. In adolescence, diagnosis of a chronic disease along with the normal personality development, bodily changes and goals for independence can give rise to anxiety, phobias and obsessive-compulsive disorders [11, 26, 29]. In addition, children and adolescents with JIA who have a reduced psychological maturity may also have a lower self-esteem and more anxiety and depression traits compared to healthy children and adolescents [6]. In other words, good self-efficacy and self-esteem can protect against psychological problems related to JIA in childhood and adolescence [10, 15].

In our study, the incidence rate of neurotic, stress-related and somatoform disorders and mood disorders seemed to be higher among females who developed JIA in adolescence (Fig. 2). Some previous studies have reported similar findings for depression and anxiety. A prospective clinical study of newly diagnosed JIA patients from 11 to 16 years of age showed significantly higher depression symptom scores in females than in males [12]. Another questionnaire study found that females with JIA (8–16 years) tended to have higher scores of anxiety and depression than did males [9]. However, a meta-analysis about behavioural problems in chronically ill children and adolescents including those with JIA showed male preponderance [30]. Males with JIA may respond to their illness with behavioural symptoms because such a behaviour is more natural for boys than girls [21, 30, 31]. Gender difference in anxiety, depression and behavioural disorders have also been observed in the general population [32].

Our study has many strengths. It covers the whole population and explores a wide spectrum of psychiatric diagnoses in all newly onset JIA patients between 2000 and 2014 compared to their population controls. The Care Register of the National Institute for Health and Welfare is systematically quality controlled [33]. Logical errors are algorithmically checked, and detected errors are sent to hospitals for correction [33]. According to a quality report, the completeness of the register was found to be very good [33].

This study does not cover mild mental health problems, which are not referred to specialized care. In addition, register study may have left out healthy controls that are less sensitive to seek health services than JIA patients with a regular contact with health care. Therefore, mental health problems might not arise either. Parental concern about the child’s active disease can also lead to increased use of mental health services in JIA patients too. All of the above may cause bias when comparing psychiatric morbidity between patients and controls. The follow-up time might not have been long enough for very young JIA patients from recent years to have time to receive a psychiatric diagnosis.

Through our research, we have received new information about the age at JIA onset and gender influence on psychiatric morbidity.

## Conclusion

In summary, patients with JIA have psychiatric diagnoses more frequently than do controls, especially female patients. Age at onset of JIA might affect the risk of psychiatric illness.

## Data Availability

All data analysed during this study are included in this published article.

## Abbreviations

JIA: Juvenile idiopathic arthritis
ICD-10: Codes of the International Classification of Diseases 10th Edition
ILAR: International League of Associations for Rheumatology
SII: The Social Insurance Institution
DMARD: Disease-modifying antirheumatic drug
HR: Hazard ratio
Cl: Confidence interval
SD: Standard deviation
IQR: Interquartile range
IRR: Incidence rate ratio
